# Erratum: characterization of iGABASnFR2 for *in vivo* mesoscale imaging of intracortical GABA dynamics

**DOI:** 10.1117/1.NPh.12.4.049801

**Published:** 2025-12-19

**Authors:** Edris Rezaei, Setare Tohidi, Mojtaba Nazari, Javad Karimi Abadchi

**Affiliations:** a University of Lethbridge, Canadian Centre for Behavioural Neuroscience, Department of Neuroscience, Lethbridge, Alberta, Canada; b McGill University, Douglas Research Centre, Department of Psychiatry, Montreal, Quebec, Canada

## Abstract

The erratum lists minor corrections to the originally published article. These corrections improve clarity and accuracy in the published article and do not alter any experimental outcomes, statistical conclusions, or interpretations.

This article [*Neurophotonics* **12**(3), 035006 (2025), DOI: 10.1117/1.NPh.12.3.035006] was originally published on 13 August 2025 with small inconsistencies that resulted from earlier drafts of the manuscript. The authors sincerely apologize for this oversight. The corrected errors, listed below, are minor reporting items only and do not affect the results, analyses, or conclusions of the study in any way.

1. Typographical correction: The control construct was mistakenly typed as “csGFP”; it was replaced with the correct name throughout: “cpSFGFP”
2. Section 2.7 — the term “C2” was removed; it was unintentionally carried over from an earlier draft and does not apply to the final experimental paradigm.
3. Section 2.11 — the phrase “between 31 and 74 Hz using a second-order Butterworth filter” was removed; this phrase referred to an early version of the preprocessing pipeline that was not used in the final analysis
4. Figure 2 Caption
  a. The color descriptions for “red” and “black” were reversed in the originally published version.
    • Original: Contralateral responses are shown in black, and ipsilateral responses are shown in red.
    • Corrected: Contralateral responses are shown in red, and ipsilateral responses are shown in black.
  b. The statistical reporting portion of the caption was removed (“Statistical analysis (two-way ANOVA): A two-way ANOVA revealed…”); this section remained from a previous draft. After remaking Figure 2 and reanalyzing the data, the correct ANOVA results were incorporated into the main text as originally published, but the older values were accidentally left in the caption.

These corrections improve clarity and accuracy in the published article and do not alter any experimental outcomes, statistical conclusions, or interpretations.

The errors were corrected on 4 December 2025.

